# Interference competition between wolves and coyotes during variable prey abundance

**DOI:** 10.1002/ece3.7153

**Published:** 2021-01-11

**Authors:** Tyler R. Petroelje, Todd M. Kautz, Dean E. Beyer, Jerrold L. Belant

**Affiliations:** ^1^ Global Wildlife Conservation Center College of Environmental Science and Forestry State University of New York Syracuse NY USA; ^2^ Wildlife Division Michigan Department of Natural Resources Marquette MI USA

**Keywords:** activity, coyote, diet, interference competition, space use, wolf

## Abstract

Interference competition occurs when two species have similar resource requirements and one species is dominant and can suppress or exclude the subordinate species. Wolves (*Canis lupus*) and coyotes (*C. latrans*) are sympatric across much of their range in North America where white‐tailed deer (*Odocoileus virginianus*) can be an important prey species. We assessed the extent of niche overlap between wolves and coyotes using activity, diet, and space use as evidence for interference competition during three periods related to the availability of white‐tailed deer fawns in the Upper Great Lakes region of the USA. We assessed activity overlap (Δ) with data from accelerometers onboard global positioning system (GPS) collars worn by wolves (*n* = 11) and coyotes (*n* = 13). We analyzed wolf and coyote scat to estimate dietary breadth (*B*) and food niche overlap (*α*). We used resource utilization functions (RUFs) with canid GPS location data, white‐tailed deer RUFs, ruffed grouse (*Bonasa umbellus*) and snowshoe hare (*Lepus americanus*) densities, and landscape covariates to compare population‐level space use. Wolves and coyotes exhibited considerable overlap in activity (Δ = 0.86–0.92), diet (*B* = 3.1–4.9; *α* = 0.76–1.0), and space use of active and inactive RUFs across time periods. Coyotes relied less on deer as prey compared to wolves and consumed greater amounts of smaller prey items. Coyotes exhibited greater population‐level variation in space use compared to wolves. Additionally, while active and inactive, coyotes exhibited greater selection of some land covers as compared to wolves. Our findings lend support for interference competition between wolves and coyotes with significant overlap across resource attributes examined. The mechanisms through which wolves and coyotes coexist appear to be driven largely by how coyotes, a generalist species, exploit narrow differences in resource availability and display greater population‐level plasticity in resource use.

## Graphical abstract

We assessed the extent of niche overlap between wolves and coyotes using activity, diet, and space use as evidence for interference competition. Our findings support interference competition between wolves and coyotes with considerable overlap across metrics examined. However, the mechanisms through which wolves and coyotes coexist appear driven largely by how coyotes, a generalist species, exploit narrow differences in resource availability and display greater population‐level plasticity in resource use.

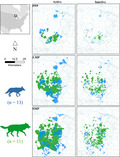

## 1 INTRODUCTION

The competitive exclusion principle posits that co‐occurring species with high resource use overlap will compete resulting in exclusion when resources are limited (Gause, 1934; Hardin, 1960). Intermediate to exclusion, resource competition can reduce fitness of individuals and result in a reduction of species abundance (Fedriani et al., 2000). Interference competition occurs where two species have similar resource requirements that are concentrated or limited and one species is dominant (e.g., kleptoparasitism, territory displacement; Case & Gilpin, 1974). Described as an active form of competition, interactions between individuals often result in the subordinate species realizing some cost (Schoener, 1983) such as loss of space (Tannerfeldt et al., 2002), reduction in time active (Hayward & Slotow, 2009), or loss of life (e.g., intraguild predation; Polis et al., 1989; Sunde et al., 1999).

Reducing interactions or competition may improve fitness for one or both species experiencing interference, as seen with cape foxes (*Vulpes chama*) avoiding black‐backed jackals (*Canis mesomelas*) to reduce interspecific killing (Kamler et al., 2012). Limiting competition also may be possible through niche partitioning (Schoener, 1974). Niche partitioning can occur through natural selection where differences in morphology arise and allow adaptation of two otherwise competing species to fill niches that are functionally different (Wilson, 1975). Ecologically, altering foraging time or effort can facilitate niche partitioning and reduce interspecific contact (Toweill, 1986). Several species of bats, similar in body size and prey selection, coexist using temporal segregation (Swift & Racey, 1983). In addition to temporal segregation, two species occupying a similar niche may exhibit spatial or dietary differentiation, or specialization, that can reduce competition and allow coexistence (Schoener, 1974). However, as prey availability varies temporally, degree of competition may also vary, changing the intensity of resource partitioning (Major & Sherburne, 1987). In field studies, interference competition is often inferred spatially (e.g., arctic fox (*Alopex lagopus*) that are excluded from red fox (*Vulpes vulpes*) territories; Tannerfeldt et al., 2002) and by measuring resource use overlap (e.g., dietary overlap among bobcats (*Lynx rufus*), coyotes (*Canis latrans*), and gray fox (*Urocyon cinereoargenteus*); Fedriani et al., 2000).

Wolves (*Canis lupus*) and coyotes are sympatric across most of their ranges in North America (Arjo & Pletscher, 2004) but differ in body size (wolves 18.0–55.0 kg [Mech, 1974]; coyotes 9.1–14.7 kg [Bekoff & Gese, 2003]). Where wolves occur, coyotes may modify their distribution, behavior, and pack size to limit interspecific competition or wolf aggression (Arjo & Pletscher, 1999; Berger & Gese, 2007; Fuller & Keith, 1981; Thurber & Peterson, 1992) and coyote abundance may be suppressed as compared to wolf‐free areas (Levi & Wilmers, 2012; Smith et al., 2003). However, co‐occurring wolves and coyotes can exhibit high spatial overlap when comparing home ranges and core areas (Arjo & Pletscher, 1999; Atwood, 2006; Berger & Gese, 2007); yet previous studies have not provided a mechanism for coexistence where this spatial overlap occurs. Home range overlap does not equate to overlap in resource use, nor does use occur across a home range or core area simultaneously or homogenously. Consideration for activity and spatial segregation between these species at finer spatial and temporal scales than the home range may provide a mechanism for coexistence. In addition, diet may be important to consider as across much of eastern North America, white‐tailed deer (*Odocoileus virginianus*) are an important prey of wolves and coyotes (Arjo et al., 2002; Ballard et al., 1999), though deer age classes selected may differ between species (Arjo et al., 2002; Kautz et al., 2019; Mech & Boitani, 2003; Patterson et al., 1998). The onset of white‐tailed deer parturition provides a large influx of vulnerable prey (Petroelje et al., 2014) that exhibits immobility and hiding behavior for about 5 weeks postparturition, followed by increased mobility and social behavior (Ozoga et al., 1982). This temporal variability in deer fawn size and mobility provides a resource within both wolves and coyotes optimal prey size range (Carbone et al., 1999) and may reduce interference competition.

We quantified the degree of temporal, dietary, and spatial overlap of wolves and coyotes at the population level to estimate the potential for interference competition and identify the mechanism for how these sympatric canids coexist using accelerometer‐enabled GPS collars, scat analysis, and resource utilization functions during May–August. We hypothesized that coyotes, as the subordinate carnivore, avoid wolves through temporal differentiation. We predicted coyotes would shift activity peaks and would exhibit reduced activity as compared to wolves. We hypothesized that wolf and coyote diets differ due to body size and optimal prey size (Carbone et al., 1999; Thurber & Peterson, 1992), where coyotes select smaller prey as compared to wolves. We predicted that wolves’ diet would be mostly white‐tailed deer as they are considered ungulate specialists. We predicted coyotes, as generalist omnivores, would exhibit a more variable diet due to avoidance of wolves and exclusion from prey resources by wolves. We hypothesized that wolves, as the dominant carnivore, exclude coyotes from areas with greatest probability of occurrence by white‐tailed deer, and use those areas disproportionately more as compared to availability. Specifically, we predicted wolves, while active, would select for areas with greater adult white‐tailed deer probabilities. We predicted that coyotes, while active, would select for areas of greater snowshoe hare and ruffed grouse densities during all time periods and greater fawn probabilities shortly after deer parturition as compared to wolves. Finally, we predicted coyote resting sites (i.e., inactive sites) would be in areas of lesser probability of wolf occurrence.

## 2 METHODS

### 2.1 Study area

This study was conducted in portions of North America's northern hardwood/boreal ecosystem in Michigan's Upper Peninsula, USA (46.27°, −88.23°), and comprised about 1,000 km^2^. Property ownership consisted of commercial forest association lands (49%), privately owned lands (33%), and state forest lands (18%). Most of the study area was forested (86%) with dominant land cover types including deciduous hardwood forests, woody wetlands, and mixed forests (Appendix A, Table A1 [2011 National Land Cover Data, Jin el al., 2013]). Coyote densities were about 10 times greater (23.8 individuals/100 km^2^) than wolf densities (2.8 individuals/100 km^2^) during 2013–2015 (Kautz et al., 2019). Densities or abundance indices for other predator and prey species in the study area include American black bears (*Ursus americanus*, 25.9/100 km^2^), bobcats (*Lynx rufus*, 3.8/100 km^2^), white‐tailed deer (571/100 km^2^ [Kautz et al., 2019]), and beaver (*Castor canadensis*, 0.11 colonies/km of river [J. Belant, unpublished data]). Elevations ranged from 401 to 550 m. Monthly average May–August temperatures ranged from highs of 24.5°C during July to lows of 2.0°C during May, and average rainfall during May–August was 34.4 cm (National Oceanic and Atmospheric Administration, 2020 1981–2010 Climate Normals).

**FIGURE 1:**
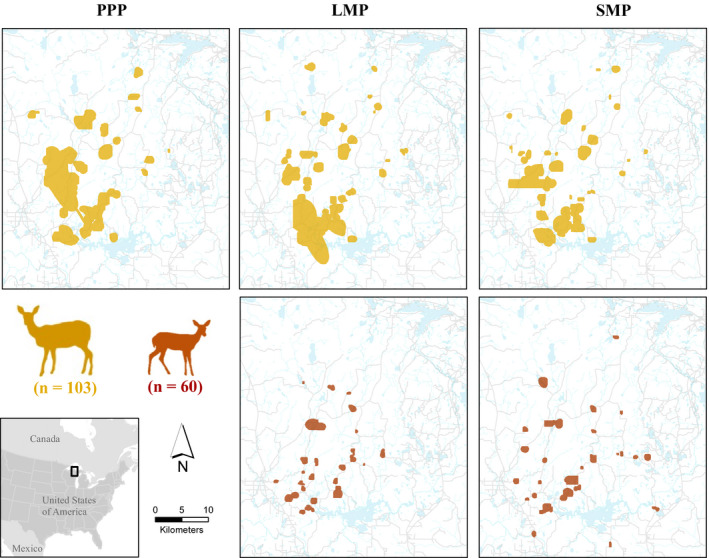
Study area showing collared adult female (dark yellow) and fawn (dark red) white‐tailed deer occurrence as semitransparent 99% occurrence distributions estimated using Brownian Bridge movement models during each time period. Also shown are roads (gray lines) and water bodies (light blue lines and polygons), Michigan's Upper Peninsula, USA, 2013–2015. Inset shows study area location (black rectangle) relative to North America

### 2.2 Capture and telemetry

We captured coyotes and wolves each spring (May–June) using No. 3 padded foothold traps (Oneida Victor) and modified MB‐750 foothold traps (modified off‐set jaws, additional swivels, and altered drag; D. Beyer, unpublished data), respectively. Additionally, we captured coyotes with relaxed locking cable restraints (Wegan et al., 2014) during February–March each year. We anesthetized coyotes and wolves with a ketamine hydrochloride (4 and 10 mg/kg, respectfully; Ketaset^®^, Fort Dodge Laboratories, Inc.) and xylazine hydrochloride (2 mg/kg; 2 mg/kg; X‐Ject E™, Butler Schein Animal Health) mixture (Kreeger et al., 2002). We fitted coyotes and wolves with a global positioning system (GPS) collar with a very high frequency (VHF) transmitter and an onboard triaxial accelerometer to record activity (Model GPS7000SU, Lotek Wireless). We programed GPS collars to acquire and store locations every 15 min from 1 May to 31 August 2013–2015. Before individuals were released at the capture site, we administered yohimbine hydrochloride (0.15 mg/kg; Hospira^©^) to reverse the effects of xylazine hydrochloride. We uploaded data weekly using ultra high frequency communication and a handheld command unit (Lotek Wireless Inc.) from a fixed‐wing aircraft. Approval for all capturing and handling procedures was through Mississippi State University's Institutional Animal Care and Use Committee (protocol 12‐012).

### 2.3 Time periods

We selected three time periods related to white‐tailed deer fawn availability to wolves and coyotes. The preparturition period (PPP, 1 May–26 May) is before the annual birth pulse of fawns occurs and only adult deer are on the landscape. The limited mobility period (LMP, 27 May–30 June) occurs when fawns are young, immobile, and within the predicted optimal prey size for coyotes beginning at fawn parturition to 35 days postparturition (Carbone et al., 1999; Ozoga et al., 1982; Petroelje et al., 2014). The social mobility period (SMP, 1 July–31 August) occurs when fawns exceed the predicted optimal prey size of coyotes (Carbone et al., 1999) and when fawn behavior switches from hiding to running with associated family groups (Nelson & Woolf, 1987). Fawns in Michigan gain on average 0.2 kg/day during their first month weighing about 9 kg by the end of LMP (Verme & Ullrey, 1984) and would reach optimal prey size for wolves during SMP. After 31 August, the fall molt begins, making it difficult to distinguish adult and fawn hair in scat samples (Adorjan & Kolenosky, 1969).

### 2.4 Estimates of prey availability

We identified white‐tailed deer, ruffed grouse (*Bonasa umbellus*), and snowshoe hare (*Lepus americanus*), a priori, as prey that may be important in wolf and coyote diets as they appeared to be dominant available prey in the study area (D. Beyer, unpublished data) and within the optimal prey size range (Carbone et al., 1999). We used snowshoe hare pellet counts to estimate hare density and grouse drumming surveys to estimate grouse density within the study area (see Appendix A, Methods).

We estimated probability of occurrence by adult female and fawn deer across the landscape using a resource utilization function (RUF; Marzluff et al., 2004) to regress the occurrence distribution (OD) of individual deer on landscape covariates thought to influence their use. To estimate ODs, we used VHF relocation data from radio‐collared adult female white‐tailed deer (*n* = 113) captured using Clover traps (Clover, 1956) and neonate fawn deer (*n* = 100) captured using vaginal implant transmitter guided searches or opportunistically during 2013–2015 (Kautz et al., 2019, 2020). We used Brownian bridge movement models (BBMM) in package “BBMM” (Nielson et al., 2013) for program R (version 3.01, R Development Core Team, 2018) to produce a 99% OD for each deer/time period (i.e., PPP, LMP, SMP) combination (Figure 1). We included adult female deer with ≥20 VHF locations or fawn deer with ≥5 VHF locations, as neonates were subject to greater predation during the first 16 weeks after birth (Kautz et al., 2019) and including only fawns with ≥20 locations would bias the average RUF toward individuals that survived. A total of 87, 89, and 94 adult female deer during PPP, LMP, and SMP, respectively, and 39 and 37 fawns during LMP and SMP, respectively, had adequate locations for analyses. The BBMM includes a term for a location error vector for estimated error of each VHF triangulation (estimated from average error triangulating known collar locations [LOAS, Ecological Software Solutions LLC.]). The BBMM also allowed specification of the maximum time step (max.lag) for motion variance to be estimated between two locations which we set to 48 hr to meet the assumption that the movement between locations was related and not random. We regressed magnitude of the OD on six landscape variables (distance to water, distance to roads, distance to edge, patch size, and land cover) thought to influence deer resource selection (Duquette et al., 2014). Because the scale of deer movement data was coarser and lacked activity data as compared to wolf and coyote data, we did not include carnivore presence to predict occurrence. We used the 2011 National Land Cover Database (NLCD, Jin et al., 2013) as a categorical assignment of land cover across the 30 × 30 m grid. We combined land covers into the following seven major classes: deciduous forest, mixed forest, evergreen forest, woody wetlands/emergent herbaceous wetlands, open water, grassland/shrub, and developed which included categories containing less than 1% of land cover (e.g., urban, agriculture, and barren; Appendix A, Table A1). We calculated landscape metrics for each cell including patch size and distance to edge (NLCD, Jin et al., 2013), distance to road (Michigan Geographic Framework, all roads v17a), and distance to water (Michigan Geographic Framework, hydrography lines v17a) in ArcMap 10.3 (Environmental Systems Research Institute) and Geospatial Modeling Environment (Beyer, 2012). Before fitting models, we used Pearson's correlation to determine any covariates that were related (i.e., |*r*| > 0.7) and selected and retained the one that was more ecologically relevant for further analyses.

We estimated the population‐level RUF for adult female and fawn deer from the individual RUF averaged coefficients for each age class during each time period using the equation

(1) $${\hat{\bar{\beta }}}_{i}=\frac{1}{n}\sum_{j=1}^{n}{\hat{\beta }}_{\mathrm{ij}}$$

where *n* is the number of individuals and ${\hat{\beta }}_{\mathrm{ij}}$ is the estimate of coefficient *i* for individual *j*. We estimated the variance of the population‐level coefficients using the equation

(2) $$Var\left({\hat{\bar{\beta }}}_{i}\right)=\frac{1}{n‐1}\sum_{j=1}^{n}{\left({\hat{\beta }}_{\mathrm{ij}}‐{\hat{\bar{\beta }}}_{i}\right)}^{2}$$

to include intraindividual and interindividual variation (Marzluff et al., 2004; Millspaugh et al., 2006). We then predicted probability of occurrence by adult female and fawn deer across the landscape for each period by using the scaled coefficients from each population‐level RUF and spatially derived a relative value for resource suitability for all model covariates layered over a 30 × 30 m cell grid which corresponds to the resolution of NLCD (Jin et al., 2013), the coarsest resource attribute.

**FIGURE 2:**
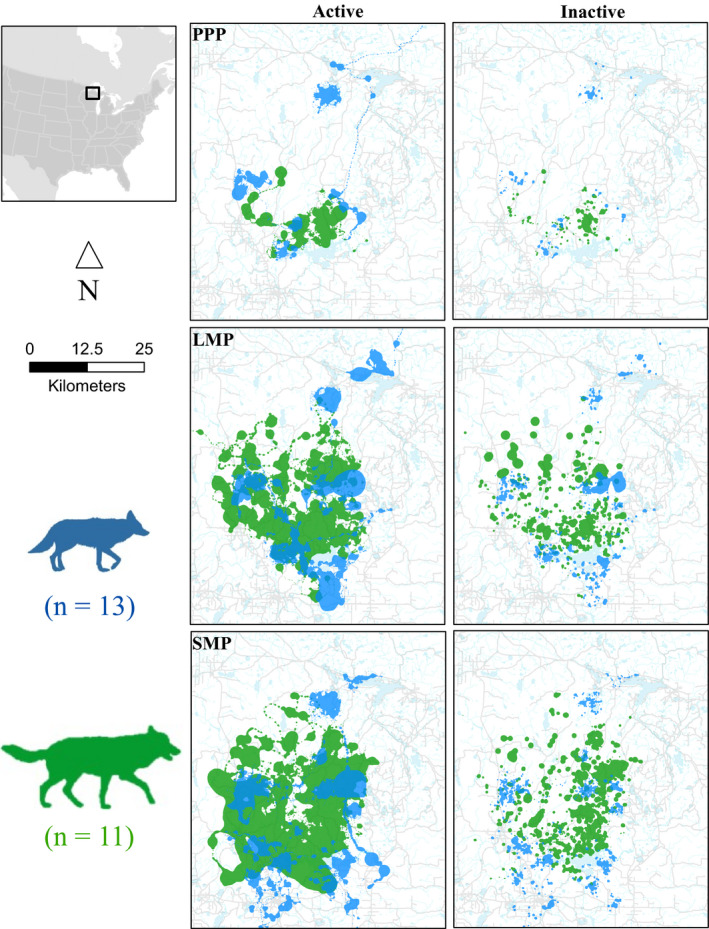
Study area showing collared wolf (green) and coyote (blue) occurrence as semitransparent 99% occurrence distributions (OD) estimated using dynamic Brownian Bridge movement models. Dark regions of OD show where occurrence overlapped with collared wolves and coyotes. Also shown are roads (gray line) and water bodies (light blue), Michigan's Upper Peninsula, USA, 2013–2015. Inset shows study area location (black rectangle) relative to North America

We used *k*‐fold cross‐validation as a measure of model fit for the RUFs of adult female and fawn deer. Following Long et al. (2009), for each fold of the cross‐validation we withheld one individual to compare model fit against and then used the remaining individuals to build a population‐level RUF. We then used that RUF to predict the probability of occurrence for each 30 × 30 m cell in the study area grid. We spatially matched and extracted the OD values from the withheld individual and the predicted values from the RUF where they overlapped on the grid. We then sorted the paired OD and RUF values based on the RUF predicted values and binned them into eight groups with equal numbers of cells in each bin. For each bin, we regressed the sum of the OD values against the sum of the RUF predicted values and then calculated the coefficient of determination (*R* ^2^) and slope of the relationship. To estimate overall model fit, we averaged *R* ^2^ and slope values across all folds (individuals) for adult female deer and fawn deer separately, where a high *R* ^2^ and a positive slope indicate good predictive power or model fit (Anderson et al., 2005; Johnson et al., 2000; Long et al., 2009).

### 2.5 Activity pattern

To assess daily activity patterns of coyotes and wolves and examine how each species partitions times of activity, we used accelerometers onboard GPS collars. Accelerometers measured gravitational acceleration four times per second along two axes (*x* and *y*). We programed GPS collars to store activity data on the collar averaged across 5‐min intervals. We considered a collared individual active when summed accelerometer readings were ≥30.7 (Petroelje et al., 2020) and subset the 5‐min intervals to observations of active intervals only. We used a one‐tailed *t* test with unequal variances to assess if coyotes, the subordinate species, were active less of the time as compared to wolves, the dominate competitor (Hayward & Slotow, 2009). We estimated the measure of mean daily (24‐hr) overlap of activity between coyotes and wolves using the active 5‐min intervals and the R package Overlap (Ridout & Linkie, 2009) for each time period (i.e., PPP, LMP, and SMP). We used the coefficient of overlapping (Δ) where 0 is no overlap and 1 is complete overlap as a measure of activity pattern overlap (Linkie & Ridout, 2011; Ridout & Linkie, 2009). We used the nonparametric estimator that works with circular data recommended for small sample sizes (Ridout & Linkie, 2009). This coefficient uses minimum probability density functions, from the kernel density estimation, for both species at each time interval to estimate the area under the curve as a measure of overlap (Linkie & Ridout, 2011).

### 2.6 Scat collection and diet analysis

We collected wolf and coyote scats opportunistically throughout the study area while driving along roads or performing other field activities during 1 May–31 August 2013–2015. We collected scats in plastic bags and labeled each with sample location, date collected, associated tracks present, and species. We used scat size and shape, and associated tracks to identify species of the deposited scat (Green & Flinders, 1981; Mech, 1970; Prugh & Ritland, 2005; Thompson, 1952). We excluded scats without associated tracks that were >28.1 and <29.0 mm as these were above the 3rd quantile for coyotes and below the 1st quantile for wolves and could therefore not be identified to species (Petroelje et al., 2019). We washed collected scats in double layered nylons and oven dried contents so all that remained was feathers, hair, bone fragments, seeds, and vegetation (Johnson & Hansen, 1979). Once contents were dried, we identified prey items including white‐tailed deer (adult or fawn; Adorjan & Kolenosky, 1969), snowshoe hare, ruffed grouse, Rodentia, seeds, and other (which included other avian species, unknown species, vegetation, and invertebrates) based on hair coloration, scale pattern, and length (Adorjan & Kolenosky, 1969; Mathiak, 1938; Spiers, 1973; Wallis, 1993). We recorded the proportion of each prey item in each scat using a 1 × 1 cm grid to estimate the percent volume of each item.

We assessed if coyote's diet contained greater volumes of deer fawns, grouse, and snowshoe hare compared to wolves using an analysis of variance. We calculated dietary breadth (*B*) and food niche overlap (*α*) for each species during each time period using Pianka’s (1973) formulas:

(3) $$B=1/\left(\sum p_{i}^{2}\right)$$

(4) $$\alpha =\sum \left(p_{i}q_{i}\right)/\sqrt{\sum p_{i}^{2}\sum q_{i}^{2}}$$

where *p_i_* is the proportion of food item *i* in the diet of predator *p* and *q_i_* is the proportion of food item *i* in the diet of predator *q*.

### 2.7 Space use

Population‐level resource selection assumes that individuals select habitats similarly (Thomas & Taylor, 2006). However, Alldredge et al. (1998) suggested this assumption is rarely met and individual variation is important for population‐level inference, especially if exclusion is occurring. Thus, we analyzed coyote and wolf location data with a Design III approach using individuals as replicates, accounting for individual‐level variation, to assess population‐level use (Thomas & Taylor, 2006). We used RUFs to relate the OD of individual wolves and coyotes to covariates thought to influence resource use.

**FIGURE 3:**
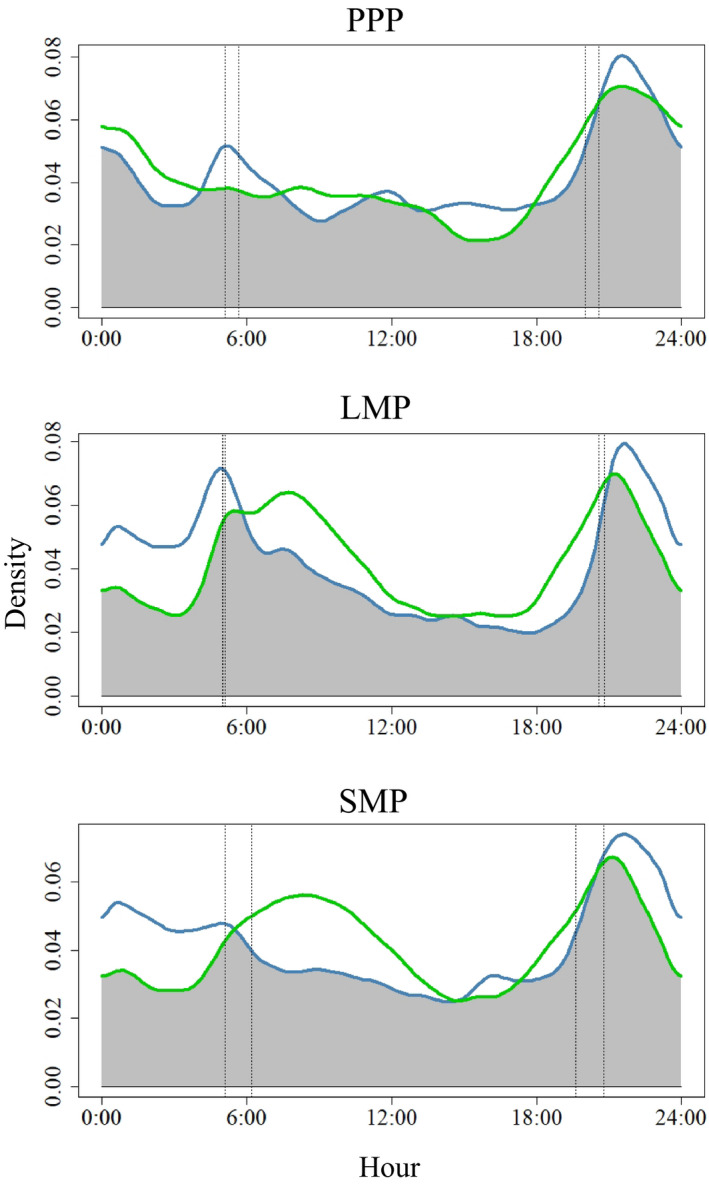
Activity patterns of wolves (green line) and coyotes (blue line) fitted with a kernel density plot showing earliest and latest sunrise and sunset (vertical dashed lines) and overlap (shaded gray) used to calculate activity overlap (Δ) during three time periods related to white‐tailed deer: preparturition period (a, 1–26 May; Δ = 0.92), fawn limited mobility period (b, 27 May–30 June; Δ = 0.86), and fawn social mobility period (c, 1 July–31 August; Δ = 0.86), Michigan's Upper Peninsula, USA, 2013–2015

To generate each OD, we used 15‐min GPS relocations ($\bar{x}$ = 1,595.7/OD) from collared wolves and coyotes collected during 1 May–31 August 2013–2015. To identify the activity state of an individual at each GPS location, we used activity data collected from accelerometers and assigned each 15‐min location as active if the nearest 5‐min activity interval was ≥30.7 (gravitational acceleration, unit‐less), otherwise we considered the location as inactive (Petroelje et al., 2020). For each collared individual, we used a dynamic Brownian bridge movement model (dBBMM; Kranstauber et al., 2017) within the package “move” for program R (version 3.01, R Development Core Team, 2018) to generate a 99% OD across a 30 × 30 m grid for all inactive (i.e., sleeping, resting) and all active (i.e., traveling, foraging) GPS relocations for each time period (i.e., PPP, LMP, and SMP; Figure 2). The dBBMM offers improvements over traditional utilization distribution estimators (e.g., fixed‐kernel estimators) as it accounts for temporal autocorrelation by using the time and distance between locations and assumes movement between locations is random, modeled as a conditional random walk, which is likely given 15‐min GPS relocations. The dBBMM estimates Brownian motion variance ($\sigma_{m}^{2}$) which varies along the GPS path via a sliding window to account for changes in movement behavior (Kranstauber et al., 2017). We selected a window of 23 locations (5.75 hr) and a margin of five locations to estimate $\sigma_{m}^{2}$ as wolves and coyotes displayed similar crepuscular activity patterns during each time period (Figure 3). We generated ODs for each individual wolf or coyote during each time period (i.e., PPP, LMP, SMP) and each activity level (active or inactive), resulting in six ODs per individual, and considered the 99% OD as the outer boundary of area available to each wolf and coyote (Thomas et al., 2006).

We used linear models (Marzluff et al., 2004) to regress the occurrence probability within each grid cell (i.e., height of the OD) on nine prey or landscape covariates to estimate the relative importance of each covariate for wolves and coyotes as a measure of space use to compare overlap. We included probability of occurrence for both adult female and fawn white‐tailed deer as well as ruffed grouse and snowshoe hare densities within each grid cell as prey that may influence coyote and wolf use. Additionally, we included the same 30 × 30 m grid of landscape covariates calculated for white‐tailed deer RUFs which included land cover, patch size, distances to nearest road, water, and land cover edge. For each coyote RUF, we also included the population‐level predicted probability of occurrence for wolves in each grid cell as a measure of avoidance. Before fitting models, we used Person's correlation to determine any covariates that were related (i.e., |*r*| > 0.7) and selected and retained the one that was more ecologically relevant for further analyses.

To estimate a population‐level RUF, we calculated standardized mean parameter estimates for each species during each activity level and time period using Equation (1) and then calculated the conservative population‐level variance using Equation (2) assuming the individuals were selected randomly from the population (Marzluff et al., 2004; Millspaugh et al., 2006). We set *α* = 0.05 for all population‐level RUFs for inference. This is conservative due to small sample size of fewer than 30 individual coyotes and wolves. To assess model fit, we used k‐fold cross‐validation of wolf and coyote RUFs following procedures used for white‐tailed deer.

## 3 RESULTS

### 3.1 Capture and telemetry

We captured and collared 19 coyotes (15 females, four males) and 12 wolves (five females, seven males). Coyotes and wolves wore collars for 102.9 (*SD* = 46.7) and 93.2 (*SD* = 24.1) days on average, respectively. Collars collected a total of 129,256 ($\bar{x}$ = 8,617.1, *SD* = 2,762.0) and 107,328 ($\bar{x}$ = 8,944.0, *SD* = 2,317.0) locations for coyotes and wolves, respectively. We recovered location and activity data from 13 coyotes (11 females, 2 males) and 11 wolves (five females, six males) for analyses; no coyotes or wolves used in analyses were collared for >1 year. Social status of individual wolves was unknown as the forested environment limited our inferences, though all individuals used in analyses were resident adults. Collared wolves represented each of the four packs within the study area. Two wolves collared from each of two packs were analyzed separately.

### 3.2 Estimates of prey availability

**FIGURE 4:**
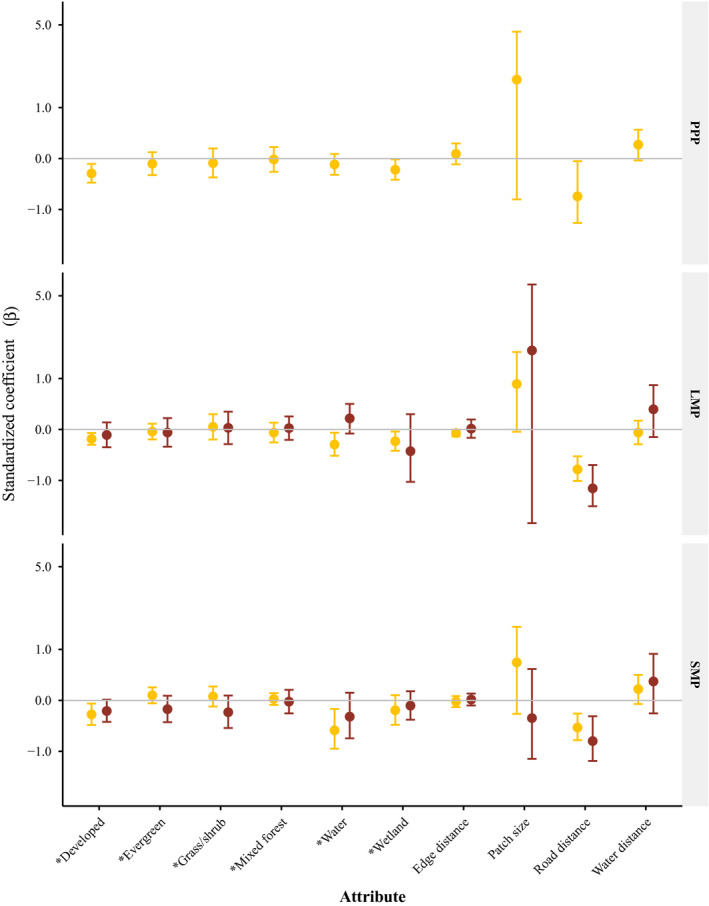
Population‐level resource utilization functions standardized coefficients (*β*) with 95% confidence intervals, for adult female (yellow) and fawn (dark red) white‐tailed deer. Land cover covariates (*) indicate selection relative to the reference value of deciduous land cover, the most common land cover on the landscape. The three time periods related to white‐tailed deer availability include preparturition period (PPP, 1–26 May), fawn limited mobility period (LMP, 27 May–30 June), and fawn social mobility period (SMP, 1 July–31 August), Michigan's Upper Peninsula, USA, 2013–2015

We used the unstandardized population‐level RUF for each deer age class and time period to develop a spatial reference for predicted deer occurrence across the 30 × 30 m grid. Adult female deer occurrence during PPP was negatively related to distance to road (*β* = −0.701, CI = −1.357 to −0.045, *p* < .036; Figure 4). During LMP, adult female deer occurrence was negatively related with distance to roads (*β* = −0.746, CI = −1.012 to −0.481, *p* < .001) and distance to edge (*β* = −0.062, CI = −0.121 to −0.004, *p* = .037). During LMP, fawn deer occurrence was also negatively related to distance to roads (*β* = −1.204, CI = −1.753 to −0.654, *p* < .001). During SMP, adult female and fawn deer occurrence was negatively related with distance to roads (*β* = −0.487, CI = −0.743 to −0.230, *p* < .001 and *β* = −0.763, CI = −1.249 to −0.277, *p* = .003, respectively). Model fit was generally good for fawns with a positive slope and *R* ^2^ > 0.45, but model fit for adult female deer was more variable with positive slopes during LMP and SMP and only during LMP was *R* ^2^ > 0.45 (Appendix B, Table B2).

### 3.3 Activity pattern

**FIGURE 5:**
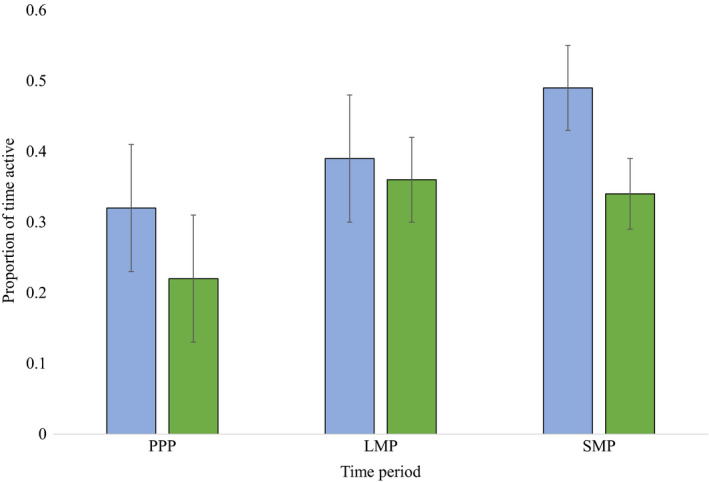
Proportion of time spent active by wolves (green) and coyotes (blue) with standard deviation shown as error bars during three time periods related to white‐tailed deer: preparturition (PPP, 1–26 May), fawn limited mobility period (LMP, 27 May–30 June), and fawn social mobility period (SMP, 1 July–31 August), Michigan's Upper Peninsula, USA, 2013–2015

Mean proportion of time spent active generally increased for both species across time periods (Figure 5). During PPP, LMP, and SMP, proportion of time spent active was 0.32 (*SD* = 0.09), 0.39 (*SD* = 0.09), and 0.49 (*SD* = 0.06) for coyotes and 0.22 (*SD* = 0.09), 0.36 (*SD* = 0.06), and 0.34 (*SD* = 0.05) for wolves, respectively. Proportion of time active between wolves and coyotes did not differ during PPP or LMP, however during SMP coyotes were more active than wolves (*p* < .01). Mean daily activity overlap for coyotes and wolves was greater than 0.86 across time periods (Table 1) though it was greatest during PPP (Δ = 0.92). Two activity peaks, one near dawn and one near dusk, were detected for both canids though wolves lacked an activity peak during dawn hours in PPP and were often more active several hours following sunrise compared to coyotes (Figure 3).

**TABLE 1:** Summary of wolves and coyotes overlap for each resource metric examined (i.e., activity, diet, and space use)

Resource metric	Time period			
	PPP	LMP	SMP	All time periods
Activity pattern^a^	0.92	0.86	0.86	0.88
Diet^b^	0.94	0.89	0.85	0.89
Spatial^c^				
Active	1.00	1.00	1.00	1.00
Inactive	1.00	1.00	1.00	1.00

Note

Though not directly comparable between resource metrics, all measures of overlap examined were high between wolves and coyotes across time periods related to white‐tailed deer: preparturition period (1–26 May), fawn limited mobility period (27 May–30 June), and fawn social mobility period (1 July–31 August), Michigan's Upper Peninsula, USA, 2013–2015.

^a^ Activity overlap (Δ = 0–1; Ridout & Linkie, 2009).

^b^ Food niche overlap (*α* = 0–1; Pianka, 1973).

^c^ Proportion of the 14 resource coefficients from resource utilization functions where use was not divergent in the direction (±) of occurrence between wolves and coyotes at the population level.

### 3.4 Scat collection and diet analysis

We collected 522 and 518 scats initially classified as coyote or wolf, respectively. Diameter of scats with confirmed coyote tracks ($\bar{x}$ = 25.2 mm, *SD* = 4.4 mm) was smaller (Welch two‐sample *t* test [*H*_a_ < 0], *p* < .01) than those from wolves ($\bar{x}$ = 33.3 mm, *SD* = 6.1 mm). We determined 377 and 305 scats to be coyote or wolf, respectively, identified by tracks or scat diameter and contained associated collection date which were used in diet analyses. Coyote scats contained 3.1 times and 1.5 times greater volumes of hare ($\bar{x}$ = 5.31%, *SD* = 3.95%, *p* < .01) and rodents ($\bar{x}$ = 23.4%, *SD* = 3.54%, *p* = .02), respectively, and 1.5 times lesser volumes of adult deer ($\bar{x}$ = 27.7%, *SD* = 4.54%, *p* < .01) compared to wolf scats. Volumes of grouse (*p* = .25) and fawns (*p* = .41) did not differ in wolf and coyote scats. Though food niche overlap varied among time periods (Table 1), it exceeded 0.85 each season and was greatest during PPP (*α* = 0.94). Dietary breadth (*B*) varied for coyotes and wolves by time period (Appendix B) but in general coyotes (*B* = 3.44–4.90) had a wider dietary breadth than wolves (*B* = 3.09–3.91). Dietary breadth was greatest for coyotes during LMP (*B* = 4.90) the same season it was least for wolves (*B* = 3.09).

### 3.5 Space use

Resource utilization functions for each species, activity level, and time period contained considerable variation among individuals; however, population‐level RUFs consistently showed greater variation in selection of resource attributes by coyotes compared to wolves (Figures 7 and 8). Though some individual wolves and coyotes selected for resource attributes similarly (Appendix B, Table B1), at the population level, few resources were selected for by all individuals. Greater variability in resource use was observed in coyotes during all time periods and activity levels except during PPP while inactive where selection for some resource attributes had greater variability for wolves. Model fit was inconsistent for wolves, all but one slope was positive and *R* ^2^ values ranged from 0.14 to 0.62. Model fit was more consistent for coyotes with all slopes positive except for one and *R* ^2^ values ranged from 0.29 to 0.53 (Appendix B, Table B2).

At the population level, wolf occurrence was not influenced by adult female deer occurrence during any time period while active or inactive. However, active wolf occurrence was positively related to hare densities (*β* = 0.028, CI = 0.003–0.054, *p* = .03) during LMP and negatively related to grouse densities (*β* = −0.035, CI = −0.058 to −0.012, *p* = .01) during PPP. During LMP, while active and inactive, wolf occurrence was negatively related to distance to edge (*β* = −0.023, CI = −0.039 to −0.008, *p* < .01 and *β* = −0.005, CI = −0.009 to −0.001, *p* = .02, respectively) similar to white‐tailed deer RUFs. During SMP, active wolf occurrence was inversely related to distance to roads and RUFs included a greater number of wolves with a positive relationship with adult female deer occurrence.

Population‐level coyote occurrence was not associated with hare or grouse densities while active or inactive. Probability of occurrence by adult female deer (which was highly correlated to occurrence of fawn deer, >0.89) also did not influence coyote occurrence at the population level during any time period or activity level (Figures 7 and 8). Population‐level coyote occurrence was not influenced by probability of wolf occurrence during any time period while active or inactive.

## 4 DISCUSSION

Wolves and coyotes exhibited considerable overlap in all metrics of resource use examined (Table 1). The greatest divergence was identified within diel activity patterns, then diet, followed by spatial partitioning during periods of activity and inactivity. Given the considerable overlap in all resource metrics, coyotes may experience interference competition by wolves; however, the combination of greater plasticity in activity, diet, and space use by coyotes likely allowed coexistence with wolves in this system.

Our prediction that coyotes may avoid wolves by altering timing of their active periods and decrease activity within those periods was not supported across time periods as activity overlap was high and coyotes were not less active than wolves (Figure 5). Wolf and coyote activity was predominantly crepuscular, with substantial overlap during all time periods as found previously (Arjo & Pletscher, 1999); however, wolves lacked a dawn activity peak during PPP when coyotes did not. The proportion of time spent active for both species generally increased across time periods, but during SMP coyotes were more active than wolves. Temporal partitioning can be used to reduce aggression when interference competition exists (Litvaitisi, 1992), though other canids exhibiting interference competition also lacked temporal partitioning (e.g., coyotes and kit fox [*Vulpes macrotis*; Kozlowski et al., 2008], coyotes and swift fox [*Vulpes velox*; Kitchen et al., 1999]). Predators are often thought to follow activity patterns of their prey, (Curio, 1976) and though both canids were most active during crepuscular periods, coyotes may not need to avoid wolves through temporal partitioning if spatial partitioning is sufficient to limit interference competition. It also is possible that temporal partitioning does not occur during summer with reduced wolf space use due to denning and pup rearing (Arjo & Pletscher, 1999). We only examined activity during summer (i.e., May–August) and greater overlap between wolves and coyotes may occur during winter months when prey is more limited (Arjo et al., 2002) and may result in temporal partitioning to reduce interference competition not identified here.

**FIGURE 6:**
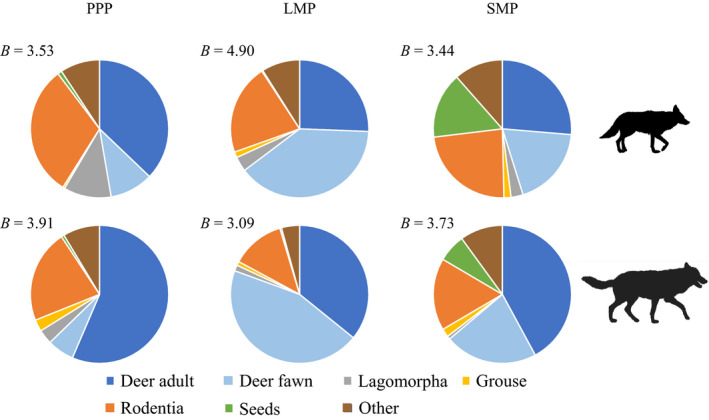
Percent of prey items identified in wolf and coyote scats during three time periods related to white‐tailed deer: preparturition (PPP, 1–26 May), fawn limited mobility period (LMP, 27 May–30 June), and fawn social mobility period (SMP, 1 July–31 August). Dietary breadth is shown for each time period and species (*B*; Pianka, 1973), Michigan's Upper Peninsula, USA, 2013–2015

Though wolves and coyotes differ in body size, and thus predicted optimal prey size (Carbone et al., 1999), dietary overlap was high during all periods (Figure 6). However, coyotes consumed greater volumes of smaller prey items than wolves. These patterns are similar to what was observed in Northwestern Montana, USA (Arjo et al., 2002) and Ontario, Canada (Benson et al., 2017) where wolf diets consistently included larger prey items as compared to coyotes. During LMP, when wolves had the narrowest dietary breadth (*B* = 3.0), coyotes exhibited the greatest dietary breadth (*B* = 4.9), apparently a result of coyotes selecting for a greater diversity of prey items not selected for by wolves. Further, wolves consistently had greater amounts of deer in their diet compared to coyotes which is expected for an obligate carnivore and ungulate specialist (Paquet & Carbyn, 2003), though deer (adult and fawns) still represented the greatest proportion of any prey for coyotes across time periods. We predicted that coyotes would select for smaller prey items based on their predicted optimal prey size (Carbone et al., 1999), and rodents and hare were found in greater volumes in coyote scat as compared to wolves. However, deer fawns and grouse found in diets of coyotes and wolves did not differ by volume in scats. Though rodents consistently represented a greater proportion of the coyote diet compared to wolves, greater differentiation would likely have been observed if prey remains of Rodentia in scat were identified to genus as beaver can be an important food resource for wolves (Mech & Peterson, 2003) and coyotes are reported to consume a variety of small mammals (Bekoff, 1977).

**FIGURE 7:**
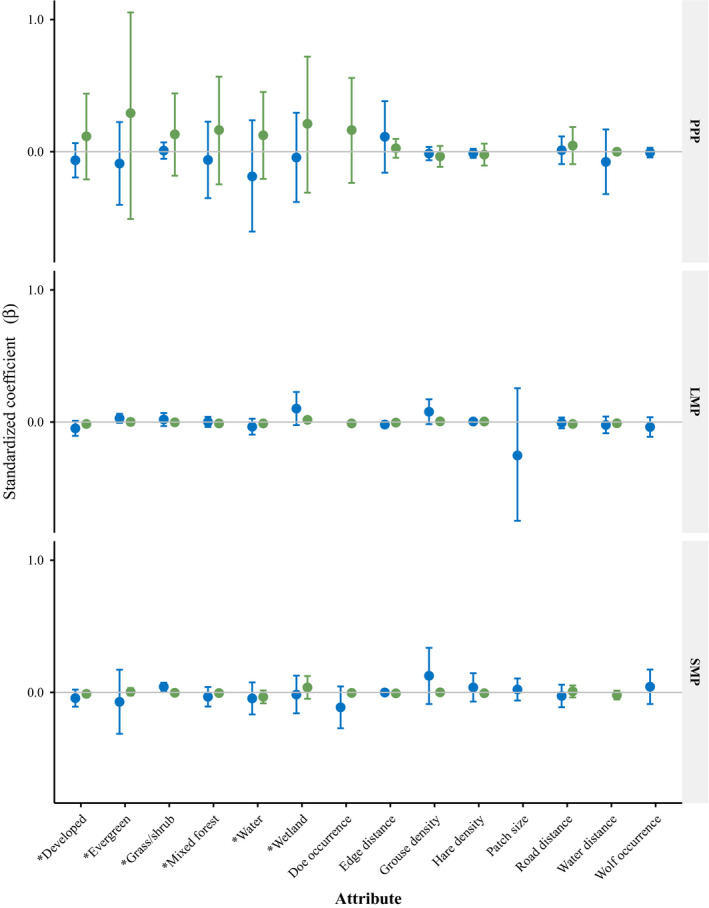
Population‐level resource utilization functions standardized coefficients (*β*) with 95% confidence intervals, for inactive wolves (green) and coyotes (blue). Land cover covariates (*) indicate selection relative to the reference value of deciduous land cover, the most common land cover on the landscape. The three time periods related to white‐tailed deer availability include preparturition (PPP, 1–26 May), fawn limited mobility period (LMP, 27 May–30 June), and fawn social mobility period (SMP, 1 July–31 August), Michigan's Upper Peninsula, USA, 2013–2015

**FIGURE 8:**
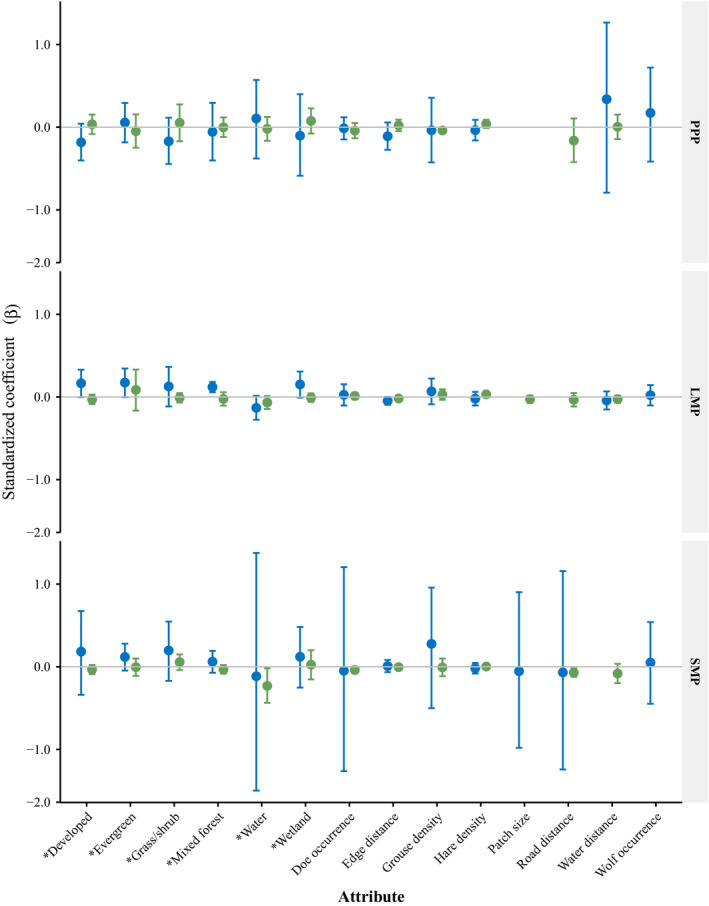
Population‐level resource utilization functions standardized coefficients (*β*) with 95% confidence intervals, for active wolves (green) and coyotes (blue). Land cover covariates (*) indicate selection relative to the reference value of deciduous land cover, the most common land cover on the landscape. The three time periods related to white‐tailed deer availability include preparturition (PPP, 1–26 May), fawn limited mobility period (LMP, 27 May–30 June), and fawn social mobility period (SMP, 1 July–31 August), Michigan's Upper Peninsula, USA, 2013–2015

We found limited evidence for spatial segregation between wolves and coyotes (Figures 7 and 8). Similarly, Berger and Gese (2007) found no evidence of spatial segregation between wolves and coyotes and Arjo and Pletscher (2004) found similar habitats were selected for by wolves and coyotes. During LMP, coyotes exhibited the widest dietary breadth and wolves the narrowest dietary breadth, suggesting that though spatial segregation was not occurring, selection for differing prey may mediate the importance of spatial segregation seasonally. In addition, the population‐level RUFs showed greater variation in selection by coyotes as compared to wolves when active and inactive. The greater variation observed in coyotes was likely due to more generalist behavior and their subordinate responses to wolves as seen in other populations (Arjo & Pletscher, 2004; Arjo et al., 2002). Resource utilization functions for individual coyotes demonstrated selection for divergent resources suggesting coyotes can employ multiple strategies to coexist with wolves at fine spatial scales (Appendix B, Table B1). This is important to consider when characterizing population‐level resource selection as individual variation may be greater (Marzluff et al., 2004), and potentially important, especially in the context of interference competition. In addition to individual variation, in complex landscapes selection of single resource attributes may not provide good estimates of species presence (as indicated by many of the individual models with multiple resource attributes influencing occurrence). Although coyotes and wolves did not select for similar attributes at the population level, individual RUFs of each species included the same significant resource attributes (Appendix B, Table B1). Given our small sample size, we did not include interaction terms for resource attributes to reduce over parameterization, though further investigation of landscape complexity and resource interactions may improve our understanding of coyote avoidance of wolves especially with respect to multiple prey species interactions. However, even at the population level examining use of resource attributes with separate RUFs for active and inactive behaviors demonstrates the complexity of resource partitioning for a coyote population coexisting with wolves and how use may differ among activities (i.e., foraging, loafing). High individual variation in resource use among coyotes as manifested at the population level likely facilitates coexistence between coyotes and wolves.

Our prediction that active wolf occurrence would be positively related to adult female deer occurrence was not supported. However, during LMP adult female and fawn deer and wolf active and inactive occurrence was negatively related to distance to edge at the population level. In addition, adult female and fawn deer and active wolf occurrence during SMP was inversely related to distance to roads. Fawn white‐tailed deer use has also been found to be greater near roads in other areas of Michigan's Upper Peninsula, USA (Duquette et al., 2014), and has been suggested as a refuge by decreasing probability of encountering wolves (Gurarie et al., 2011; Muhly et al., 2011; Theuerkauf & Rouys, 2008). However, wolves sometimes use roads and trails for travel (Thurber et al., 1994; Whittington et al., 2005) and may hunt along these features as seen in Banff and Jasper National Parks, Canada, where wolves encounter rates with caribou (*Rangifer tarandus*) increased near anthropogenic linear features (Whittington et al., 2011).

We predicted active coyotes would select areas of greater probability of occurrence for fawns, snowshoe hares, and ruffed grouse. Though fawns were a large proportion of the diet of coyotes during LMP (Figure 6), we did not see increasing coyote occurrence with greater deer probability (Figures 7 and 8). Coyotes can respond functionally with respect to fawn consumption (Petroelje et al., 2014) and may not shift their space use to select for areas of high fawn use (Svoboda et al., 2019). Coyote occurrence was not positively related to hare density (Figures 7 and 8), and though hare represented a smaller proportion of the coyote diet, the lack of a spatial response suggests coyotes may have also responded functionally as hare densities declined significantly over the study period (Appendix A, Table A2). Coyote occurrence was not influenced by grouse density though we would not expect a large spatial response as grouse represented a small proportion of the diet of coyotes across time periods (Figure 6).

We predicted inactive coyote occurrence would be inversely related to wolf occurrence to avoid encounters during vulnerable activities such as loafing or sleeping, but at the population‐level RUF this prediction was not supported (Figure 7). Coyote avoidance of areas with greater wolf use has been observed in Michigan's Upper Peninsula, USA (Svoboda et al., 2019), though these areas of wolf use were reduced and intensity of use greater due to smaller home ranges resulting from scavenging on livestock carcass dumps which were not present in our study area (Petroelje et al., 2019). This variation in spatial response to wolves regionally may be explained by risk of aggressive interactions. Merkle et al. (2009) found that 79% of wolf–coyote interactions occurred at wolf‐killed carcasses and 7% of those interactions resulted in a coyote mortality; thus, avoidance of wolves may be less important where scavenging wolf kills is less common.

Predation on coyotes by wolves is often used to confirm interference competition (Arjo & Pletscher, 1999; Berger & Gese, 2007; Merkle et al., 2009; Thurber & Peterson, 1992) and can account for up to 50% of mortality for transient coyotes (Berger & Gese, 2007). Interference competition between wolves and coyotes occurs in the greater Yellowstone ecosystem where coyote densities in areas with wolves (coyotes, 0.19–0.48/km^2^; wolves, 0.01–0.06/km^2^) are reduced or limited compared to coyote densities in wolf‐free areas (0.35–0.73/km^2^; Berger & Gese, 2007). In our study area, wolf (0.03/km^2^) and coyote (0.19–0.24/km^2^) populations occur at similar densities to the greater Yellowstone ecosystem, and wolf densities appear to have been stable since 2010 (O’Neil, 2017). Individual coyotes were only collared for a single summer and fall and we did not record any wolf predation of collared coyotes; the only documented causes of mortality were human caused and only one uncollared coyote was found killed by wolves at a deer predation site during the study (J. Belant, unpublished data). However, aggressive interactions of wolves and coyotes likely decrease over time when wolves recolonize (Merkle et al., 2009), and wolves have been reestablished at moderate densities in the western Upper Peninsula of Michigan since the late 1990s (Beyer et al., 2009). Additionally, our study area was mostly forested, in contrast to more open habitats of the western United States, which is likely to influence visible distance, scent dispersion, and spatial overlap between wolves and coyotes. Greater habitat complexity can result in lesser competition by reducing niche overlap (Levins, 1979) and reductions in scent dispersion in complex habitats increases search times for detection dogs (Leigh & Dominick, 2015) and likely reflect conditions experienced by wolves and coyotes.

Alternatively, Crimmins and Deelan (2019) suggest that in areas where white‐tailed deer are a main prey source, as in this study, coyotes are less likely to scavenge wolf kills as they are capable of killing adult deer, potentially reducing conflict in systems without large bodied ungulate resources. They found no evidence that increasing wolf populations were limiting coyote abundance in Wisconsin, USA, which shares many similarities with our study area in Michigan's Upper Peninsula, USA, though lesser wolf densities may also be important in facilitating coexistence in that region. Though deer were the greatest shared prey for wolves and coyotes in this study, based on the generalist nature of deer as supported by the adult female and fawn RUFs, it seems unlikely that deer present a concentrated prey source during the study period. Further, during this time fawns are of size to be consumed in a single meal or easily transported which reduces likelihood of scavenging and adult deer are difficult to capture.

## 5 CONCLUSIONS

Interference competition suggests that dominant species can suppress or exclude subordinate competitors where resource use overlap is high (Case & Gilpin, 1974). Diet, space use, and activity of coyotes overlapped substantially with wolves, and thus, coyotes may experience interference competition from dominant wolves. However, exclusion of coyotes by wolves appeared to be mediated through greater generalist behavior by coyote's selection of smaller prey, greater variation in prey selection and spatial partitioning when active and inactive, and greater time spent active during some time periods. This fine scale resource partitioning may be the mechanism for coexistence in other areas where coyote abundance is not suppressed by wolves. We suggest that though coyotes may experience interference competition by wolves, a stable population of coyotes, and the ability to coexist in a heavily forested environment occurred through ecological plasticity of coyotes’ diet, space use, and activity. Where interference competition occurs, the subordinate species may be able to avoid exclusion through greater generalist behavior and facilitate coexistence. Thus, communities may support greater densities or numbers of species of competitors than expected if flexibility in resource use is sufficient to allow coexistence.

## CONFLICT OF INTEREST

The authors declare that they have no competing interests.

## AUTHOR CONTRIBUTION

**Tyler Petroelje:** Conceptualization (lead); Data curation (lead); Formal analysis (lead); Investigation (lead); Methodology (lead); Project administration (lead); Visualization (lead); Writing‐original draft (lead). **Todd M Kautz:** Conceptualization (supporting); Data curation (equal); Formal analysis (supporting); Investigation (equal); Methodology (supporting); Project administration (equal); Writing‐review & editing (supporting). **Dean E. Beyer, Jr.:** Conceptualization (equal); Funding acquisition (lead); Investigation (supporting); Project administration (supporting); Resources (equal); Supervision (equal); Writing‐review & editing (equal). **Jerrold L. Belant:** Conceptualization (equal); Formal analysis (supporting); Funding acquisition (lead); Methodology (supporting); Project administration (supporting); Resources (equal); Supervision (lead); Writing‐review & editing (lead).

## Supporting information

Appendix S1‐S2

## Data Availability

Data used in this manuscript are catalogued in the Dryad Digital Repository (available at https://doi.org/10.5061/dryad.4f4qrfj9w).

## APPENDIX A

### Methods, results, and tables for estimating ruffed grouse and snowshoe hare densities

#### Methods

##### Snowshoe hare

Following recommendations of Hodges and Mills (2008), we estimated snowshoe hare abundance from mid‐April to early May 2013–2015, following snowmelt, by counting fecal pellet groups within 1 m^2^ plots. Within each land cover class (Jin et al., 2013, Table A1), we randomly generated 200 plot locations separated by ≥50 m using ArcMap 10.3 (Environmental Systems Research Institute) and haphazardly selected sites to visit and attempted to sample ≥80 plots in each dominant land cover (>5%) and aspen (12%; *Populus tremuloides* or *P. grandidentata*; Ellenwood et al., 2015), as it is preferred winter forage for snowshoe hares (Bookhout, 1965) and differs from the dominant deciduous cover (i.e., sugar maple [*Acer saccharum*]). We sampled remaining land cover types, with ≥30 pellet plot sites in each, to identify if any were of importance for snowshoe hare (“open water” and “developed” were not sampled). At each site, we compared the land cover layer designation to the actual vegetation observed using the designations provided by Jin et al. (2013) to correctly assign each plot for land cover classification. Each plot was a 10‐cm × 10‐m rectangle, and we counted all pellets greater than 50% contained by the rectangle. We used plots that were uncleared of hare pellets prior to surveying as they do not require waiting a year before estimating hare density. These estimates may be greater than when using cleared plots if previous years pellets have not degraded (Berg & Gese, 2010; Murray et al., 2002, 2005) though uncleared plots have provided similar estimates of hare density as cleared plots (Hodges & Mills, 2008) and any bias from using uncleared plots should remain constant across years as new sites were sampled each year. Following Murray et al. (2002), we related pellet density (mean pellets/m^2^ [*x*]) to hare density (hares/19 ha [*y*]), where *y* = exp (1.112 + 1.047*(ln *x* + 1/6)). For comparison to other prey densities and to apply densities to the landscape scale, we converted hares/ha to hares/km^2^ and applied a correction factor of 1.41 to account for natural log bias produced from the transformation (Murray et al., 2002). In addition, we calculated a study area density using the weighted mean by proportion of land cover to examine trends in the hare population over time.

##### Ruffed grouse

We used 65 roadside male grouse drumming survey sites and five visits to estimate density of grouse. Surveys were conducted when wind speeds were <8 mph and there was no precipitation, as these conditions may inhibit bird activity or detection (Zimmerman and Gutiérrez, 2007). We established survey sites >1.6 km apart to ensure site independence and assumed grouse have a maximum detection radius of 550 m from each survey point (Hansen et al., 2011). We conducted surveys from late April to early May 2013–2015 at the peak of ruffed grouse drumming in the Upper Great Lakes region (Michigan Department of Natural Resources, 2012). We conducted surveys from 0.5 hr before sunrise to 5 hr after sunrise and listened for grouse drumming for 5 min at each site to assess presence/absence of grouse (Hansen et al., 2011). We used an *N*‐mixture model framework (Kery et al., 2005; Royle, 2004) which estimates detection probability and site abundance using function “pcount” within package unmarked (Fiske & Chandler, 2011) for program R (version 3.01, R Development Core Team, 2018) to estimate drumming grouse density. We used number of drumming grouse at each site, during each of the five visits, as the response data modeled as a Poisson distribution. We expected the timing of survey visits would influence detection of drumming grouse, given the seasonality of this behavior, and included survey date as a covariate of detection. We included proportion of aspen land cover (Ellenwood et al., 2015) within each site detection radius as a covariate of abundance. We used Akaike information criterion for small sample sizes (AICc) to rank models for best fit (Burnham & Anderson, 2002) to estimate grouse abundance. We considered all combinations of covariates of detection and abundance, a total of four models each year, and we considered the model with the least AICc score as the best supported model for each year. We assumed the grouse population had a 1:1 sex ratio (Gullion, 1981) and estimated the population density by doubling the estimated drumming (i.e., male) grouse abundance from the best supported *N*‐mixture model and converted this number to a density by dividing it by the total area surveyed.

#### Results

##### Snowshoe hare

We sampled 316, 413, and 448 pellet plots during 2013, 2014, and 2015, respectively. Mean pellets detected per plot ranged from 0.0 (CI = 0.0–0.7) in deciduous (excluding aspen) land covers to 5.6 (CI = 0.0–45.9) in woody wetlands (Table A2). Hare density was greatest during 2013 in aspen land cover (33.1/km^2^) and least during 2015 in deciduous hardwoods (3.5/km^2^). Hare density generally declined across years (2013–2015) when examined by all land cover types.

##### Ruffed grouse

We detected an average of 0.7, 0.4, and 0.6 drumming grouse at each site during 2013–2015, respectively. Timing of survey visit (i.e., date) influenced detection of drumming grouse during all three survey years (Table A3). *N*‐mixture models estimated detection (15.8%–33.4%) and abundance (137–178) as relatively stable across years with confidence intervals overlapping each year (Table A3). Drumming male grouse abundance estimates were doubled to estimate a population density of 5.8, 4.9, and 4.4 grouse/km^2^ during 2013–2015, respectively. In 2013, the top model included a positive relationship with proportion of aspen. No covariates of abundance were important to predicting grouse density in 2014 and 2015.

**TABLE A1:** Land cover designations modified from the national land cover database with percent land cover within study area, extracted from Jin et al. (2013), Michigan's Upper Peninsula, USA, 2011

Land cover class	Definition of designation	Cover (%)
Deciduous forest	Areas dominated by trees generally greater than 5 m tall, and greater than 20% of total vegetation cover. More than 75% of the tree species shed foliage simultaneously in response to seasonal change Aspen (*Populus tremuloides* or *P. grandidentata*) represents dominant cover for 12% of deciduous forests within the study area (Ellenwood et al., 2015)	43
Woody or emergent herbaceous wetland	Areas where forest or shrub land vegetation accounts for greater than 20% of vegetative cover and the soil or substrate is periodically saturated with or covered with water. Areas where perennial herbaceous vegetation accounts for greater than 80% of vegetative cover and the soil or substrate is periodically saturated with or covered with water	29
Mixed forest	Areas dominated by trees generally greater than 5 m tall, and greater than 20% of total vegetation cover. Neither deciduous nor evergreen species are greater than 75% of total tree cover	10
Evergreen forest	Areas dominated by trees generally greater than 5 m tall, and greater than 20% of total vegetation cover. More than 75% of the tree species maintain their leaves all year. Canopy is never without green foliage	6
Grassland/herbaceous/shrub/scrub	Areas dominated by grammanoid or herbaceous vegetation, generally greater than 80% of total vegetation. These areas are not subject to intensive management such as tilling but can be utilized for grazing. Areas dominated by shrubs; less than 5 m tall with shrub canopy typically greater than 20% of total vegetation. Includes true shrubs, young trees in an early successional stage or trees stunted from environmental conditions	5
Open water	Areas of open water, generally with less than 25% cover or vegetation or soil	4
Developed (i.e., urban, barren, pasture, agriculture)	All other areas modified by agriculture or developed land use practices such as farmed row crops, pastures, roads, and structures	3

**TABLE A2:** Mean ($\bar{x}$) pellet counts for snowshoe hare pellet plots with 95% confidence intervals (CI) by dominant land cover or species (i.e., aspen; *Populus tremuloides* or *P. grandidentata*) classification with number of sites (*n*) and estimated density (hare/km^2^) by land cover and overall study area for each year, Michigan's Upper Peninsula, USA, 2013–2015

Year	Land cover	*n*	$\bar{x}$	2.5% CI	97.5% CI	Density by land cover	Study area density^b^
2013	Aspen	34	4.0	0.0	18.7	33.1	15.4
	Deciduous^a^	52	0.2	0.0	0.7	3.9	
	Evergreen	80	4.0	0.0	16.4	20.2	
	Mixed	81	5.1	0.0	30.0	24.2	
	Woody wetland	69	3.7	0.0	19.3	22.9	
2014	Aspen	80	2.7	0.0	12.8	9.8	9.5
	Deciduous^a^	87	0.3	0.0	0.0	3.8	
	Evergreen	86	3.0	0.0	18.3	12.6	
	Mixed	81	2.3	0.0	19.0	10.3	
	Woody wetland	79	5.6	0.0	45.9	18.6	
2015	Aspen	90	0.6	0.0	6.8	5.6	6.5
	Deciduous^a^	88	0.0	0.0	0.0	3.5	
	Evergreen	83	2.3	0.0	15.0	10.5	
	Mixed	110	2.1	0.0	25.9	7.9	
	Woody wetland	77	2.6	0.0	21.2	11.5	

^a^ Excluding aspen.

^b^ Weighted mean by proportion of each land cover within the study area.

**TABLE A3:** Top N‐mixture model for ruffed grouse drumming surveys each year as determined by AICc selection including estimates of detection and abundance with 95% confidence intervals (CI), Michigan's Upper Peninsula, USA, 2013–2015

Year	Model^a^	Detection estimate (%)	Abundance estimate^b^	95% CI
2013	~date ~ asp	24.5	178	93–346
2014	~date ~ 1	15.8	151	79–1246
2015	~date ~ 1	33.4	137	92–239

^a^ N‐mixture model includes covariates of detection on the left and abundance on the right. The “date” covariate was Julian date. The null model (intercept only) is indicated as “1.” Covariates for ruffed grouse include “asp” as the proportion of aspen (*Populus tremuloides* or *P. grandidentata*) as land cover within each survey site.

^b^ Abundance estimates are for the audible area surveyed (550 m diameter with 65 sites for grouse) and only include estimates of abundance for drumming males in grouse surveys.

## APPENDIX B

Significant resource attributes from population‐level resource utilization functions (RUF) for wolves and coyotes and *k*‐fold cross‐validation results for RUFs of wolves, coyotes, and white‐tailed deer, Michigan's Upper Peninsula, USA, 2013–2015.

**TABLE B1:** Number of individuals that had significant (*α* < 0.05, confidence intervals do not include 0) positive (+) or negative (−) modeled relationship with each resource attribute from population‐level resource utilization functions for wolves and coyotes (excluding land cover covariates). Resource utilization functions were estimated for active and inactive GPS locations during three time periods related to white‐tailed deer: preparturition (PPP, 1–26 May), fawn limited mobility period (LMP, 27 May–30 June), and fawn social mobility period (SMP, 1 July–31 August), Michigan's Upper Peninsula, USA, 2013–2015

Resource attribute	Coyote												Wolf											
	Active						Inactive						Active						Inactive					
	PPP		LMP		SMP		PPP		LMP		SMP		PPP		LMP		SMP		PPP		LMP		SMP	
	+	−	+	−	+	−	+	−	+	−	+	−	+	−	+	−	+	−	+	−	+	−	+	−
Intercept	1	4	4	6	3	8	0	3	1	4	0	6	1	3	3	4	7	4	0	0	2	2	2	3
Distance to edge	0	5	2	8	4	7	1	2	2	3	1	5	2	2	0	7	3	8	0	0	0	4	0	5
Distance to road	NA	NA	NA	NA	6	5	1	2	1	4	3	3	1	3	3	4	3	8	0	0	1	3	0	5
Distance to water	3	2	4	6	2	3	NA	NA	2	1	NA	NA	1	3	1	6	2	9	0	0	0	4	0	5
Doe occurrence	2	3	5	5	6	5	NA	NA	NA	NA	2	4	1	3	3	4	3	8	0	0	2	2	2	3
Grouse density	2	3	6	4	4	7	2	1	2	3	4	2	0	4	5	2	6	5	0	0	2	2	2	3
Hare density	2	3	5	5	6	5	0	3	2	3	4	2	4	0	5	2	6	5	0	0	4	0	2	3
Patch size	NA	NA	NA	NA	5	6	NA	NA	2	3	1	5	NA	NA	1	6	NA	NA	NA	NA	NA	NA	NA	NA
Wolf occurrence	3	2	6	4	6	5	1	2	2	3	2	4	NA	NA	NA	NA	NA	NA	NA	NA	NA	NA	NA	NA

**TABLE B2:** K‐fold cross‐validation results for resource utilization functions for wolves, coyotes, adult female deer, and fawn deer during three time periods related to white‐tailed deer: preparturition (PPP, 1–26 May), fawn limited mobility period (LMP, 27 May–30 June), and fawn social mobility period (SMP, 1 July–31 August), Michigan's Upper Peninsula, USA, 2013–2015

Species	Activity	Period	Slope	*R* ^2^	Positive slope	Negative slope	Significant positive	Significant negative
Wolves	Active	PPP	−2.02E−03	0.42	2	3	1	1
		LMP	9.17E−03	0.62	10	1	8	0
		SMP	1.16E−02	0.39	8	3	4	0
	Inactive	PPP	4.93E−03	0.14	4	2	0	0
		LMP	8.86E−03	0.53	11	0	7	0
		SMP	7.30E−03	0.28	9	2	1	0
Coyotes	Active	PPP	5.15E−02	0.35	5	2	3	0
		LMP	1.43E−03	0.54	7	6	5	2
		SMP	−7.41E−05	0.32	4	9	2	1
	Inactive	PPP	6.20E−02	0.25	5	2	2	0
		LMP	5.17E−04	0.38	8	5	4	1
		SMP	6.84E−04	0.29	7	6	2	1
Adult female deer	—	PPP	−3.14E−05	0.12	54	33	0	1
		LMP	4.39E−03	0.49	72	17	41	6
		SMP	1.54E−05	0.15	52	42	4	1
Fawn deer	—	LMP	4.95E−03	0.45	28	9	15	2
		SMP	3.94E−03	0.54	34	3	20	0
