# Supplementary material for: Interference competition between wolves and coyotes during variable prey abundance
Source: Ecol Evol. 2021 Jan 11;11(3):1413–31. doi: 10.1002/ece3.7153 (PMC7863399; doi:10.1002/ece3.7153)
Supplement: Supplementary file 1 — Appendix S1‐S2 [file ECE3-11-1413-s001.docx]

**APPENDIX A**

**Methods, results, and tables for estimating ruffed grouse and snowshoe hare densities.**

**Methods**

*Snowshoe hare* – Following recommendations of Hodges and Mills (2008), we estimated snowshoe hare abundance from mid April to early May 2013–2015, following snowmelt, by counting pellet groups within 1 m^2^ plots. Within each land cover class (Jin et al 2013, Table A1), we randomly generated 200 plot locations separated by >50 m using ArcMap 10.3 (Environmental Systems Research Institute, Redlands, CA, USA) and haphazardly selected sites to visit and attempted to sample ≥80 plots in each dominant land cover (>5%) and aspen (12%; *Populus tremuloides* or *P. grandidentata*; Ellenwood et al. 2015), as it is preferred winter forage for snowshoe hares (Bookhout 1965) and differs from the dominant deciduous cover (i.e., sugar maple [*Acer saccharum*]). We sampled remaining land cover types, with ≥30 pellet plot sites in each, to identify if any were of importance for snowshoe hare (“open water” and “developed” were not sampled). At each site, we compared the land cover layer designation to the actual vegetation observed using the designations provided by Jin et al. (2013) to correctly assign each plot for land cover classification. Each plot was a 10-cm × 10-m rectangle and we counted all pellets greater than 50% contained by the rectangle. We used plots that were uncleared of hare pellets prior to surveying as they do not require waiting a year before estimating hare density. These estimates may be greater than when using cleared plots if previous years pellets have not degraded (Murray et al. 2002, Murray et al. 2005, Berg and Gese 2010) though uncleared plots have provided similar estimates of hare density as cleared plots (Hodges and Mills 2008) and any bias from using uncleared plots should remain constant across years as new sites were sampled each year. Following Murray et al. (2002) we related pellet density (mean pellets/m^2^ [*x*]) to hare density (hares/19 ha [*y*]), where *y* = exp (1.112 + 1.047*(ln *x* + 1/6)). For comparison to other prey densities and to apply densities to the landscape scale we converted hares/ha to hares/km^2^ and applied a correction factor of 1.41 to account for natural log bias produced from the transformation (Murray et al. 2002). In addition, we calculated a study area density using the weighted mean by proportion of land cover to examine trends in the hare population over time.

*Ruffed grouse* – We used 65 roadside male grouse drumming survey sites and 5 visits to estimate density of grouse. Surveys were conducted when wind speeds were <8 mph and there was no precipitation, as these conditions may inhibit bird activity or detection (Zimmerman and Gutierrez 2007). We established survey sites >1.6 km apart to ensure site independence and assumed grouse have a maximum detection radius of 550 m from each survey point (Hansen et al. 2011). We conducted surveys from late April to early May 2013–2015 at the peak of ruffed grouse drumming in the upper Great Lakes region (Michigan Department of Natural Resources 2012). We conducted surveys from 0.5 hour before sunrise to 5 hours after sunrise and listened for grouse drumming for 5 minutes at each site to assess presence/absence of grouse (Hansen et al. 2011). We used an N-mixture model framework (Royle 2004, Kery et al. 2005) which estimates detection probability and site abundance using function ‘pcount’ within package unmarked (Fiske and Chandler 2011) for program R (version 3.01, R Development Core Team 2018) to estimate drumming grouse density. We used number of drumming grouse at each site, during each of the 5 visits, as the response data modeled as a Poisson distribution. We expected the timing of survey visits would influence detection of drumming grouse, given the seasonality of this behavior, and included survey date as a covariate of detection. We included proportion of aspen landcover (Ellenwood et al. 2015) within each site detection radius as a covariate of abundance. We used Akaike Information Criterion for small sample sizes (AICc) to rank models for best fit (Burnham and Anderson 2002) to estimate grouse abundance. We considered all combinations of covariates of detection and abundance, a total of 4 models each year, and we considered the model with the least AICc score as the best supported model for each year. We assumed the grouse population had a 1:1 sex ratio (Gullion 1981) and estimated the population density by doubling the estimated drumming (i.e., male) grouse abundance from the best supported N-mixture model and converted this number to a density by dividing it by the total area surveyed.

**Results**

*Snowshoe hare* – We sampled 316, 413, and 448 pellet plots during 2013, 2014, and 2015, respectively. Mean pellets detected per plot ranged from 0.0 (CI = 0.0–0.7) in deciduous (excluding aspen) land covers to 5.6 (CI = 0.0–45.9) in woody wetlands (Table A2). Hare density was greatest during 2013 in aspen land cover (33.1/km^2^) and least during 2015 in deciduous hardwoods (3.5/km^2^). Hare density generally declined across years (2013–2015) when examined by all land cover types.

*Ruffed grouse* – We detected an average of 0.7, 0.4, and 0.6 drumming grouse at each site during 2013–2015, respectively. Timing of survey visit (i.e., date) influenced detection of drumming grouse during all 3 survey years (Table A3). N-mixture models estimated detection (15.8%–33.4%) and abundance (137–178) as relatively stable across years with confidence intervals overlapping each year (Table A3). Drumming male grouse abundance estimates were doubled to estimate a population density of 5.8, 4.9, and 4.4 grouse/km^2^ during 2013–2015, respectively. In 2013 the top model included a positive relationship with proportion of aspen. No covariates of abundance were important to predicting grouse density in 2014 and 2015.

Table A1. Land cover designations modified from the national land cover database with percent land cover within study area, extracted from Jin et al. (2013), Michigan’s Upper Peninsula, USA, 2011.

| Land cover class | Definition of designation | Cover (%) |
| --- | --- | --- |
| Deciduous forest | Areas dominated by trees generally greater than 5 meters tall, and greater than 20% of total vegetation cover. More than 75% of the tree species shed foliage simultaneously in response to seasonal change.  Aspen (*Populus tremuloides* or *P. grandidentata*) represents dominant cover for 12% of deciduous forests within the study area (Ellenwood et al. 2015). | 43 |
| Woody or emergent herbaceous wetland | Areas where forest or shrub land vegetation accounts for greater than 20% of vegetative cover and the soil or substrate is periodically saturated with or covered with water. Areas where perennial herbaceous vegetation accounts for greater than 80% of vegetative cover and the soil or substrate is periodically saturated with or covered with water. | 29 |
| Mixed forest | Areas dominated by trees generally greater than 5 meters tall, and greater than 20% of total vegetation cover. Neither deciduous nor evergreen species are greater than 75% of total tree cover. | 10 |
| Evergreen forest | Areas dominated by trees generally greater than 5 meters tall, and greater than 20% of total vegetation cover. More than 75% of the tree species maintain their leaves all year. Canopy is never without green foliage. | 6 |
| Grassland/herbaceous/shrub/scrub | Areas dominated by grammanoid or herbaceous vegetation, generally greater than 80% of total vegetation. These areas are not subject to intensive management such as tilling but can be utilized for grazing. Areas dominated by shrubs; less than 5 meters tall with shrub canopy typically greater than 20% of total vegetation. Includes true shrubs, young trees in an early successional stage or trees stunted from environmental conditions. | 5 |
| Open water | Areas of open water, generally with less than 25% cover or vegetation or soil. | 4 |
| Developed (i.e., urban, barren, pasture, agriculture) | All other areas modified by agriculture or developed land use practices such as farmed row crops, pastures, roads, and structures. | 3 |

Table A2. Mean ($\bar{x}$) pellet counts for snowshoe hare pellet plots with 95% confidence intervals (CI) by dominant land cover or species (i.e., aspen; *Populus tremuloides* or *P. grandidentata*) classification with number of sites (*n*) and estimated density (hare/km^2^) by landcover and overall study area for each year, Michigan’s Upper Peninsula, USA, 2013–2015.

| Year | Land cover | *n* | $\bar{x}$ | 2.5% CI | 97.5% CI | Density by land cover | Study area density^b^ |
| --- | --- | --- | --- | --- | --- | --- | --- |
| 2013 | Aspen | 34 | 4.0 | 0.0 | 18.7 | 33.1 | 15.4 |
|  | Deciduous^a^ | 52 | 0.2 | 0.0 | 0.7 | 3.9 |  |
|  | Evergreen | 80 | 4.0 | 0.0 | 16.4 | 20.2 |  |
|  | Mixed | 81 | 5.1 | 0.0 | 30.0 | 24.2 |  |
|  | Woody wetland | 69 | 3.7 | 0.0 | 19.3 | 22.9 |  |
| 2014 | Aspen | 80 | 2.7 | 0.0 | 12.8 | 9.8 | 9.5 |
|  | Deciduous^a^ | 87 | 0.3 | 0.0 | 0.0 | 3.8 |  |
|  | Evergreen | 86 | 3.0 | 0.0 | 18.3 | 12.6 |  |
|  | Mixed | 81 | 2.3 | 0.0 | 19.0 | 10.3 |  |
|  | Woody wetland | 79 | 5.6 | 0.0 | 45.9 | 18.6 |  |
| 2015 | Aspen | 90 | 0.6 | 0.0 | 6.8 | 5.6 | 6.5 |
|  | Deciduous^a^ | 88 | 0.0 | 0.0 | 0.0 | 3.5 |  |
|  | Evergreen | 83 | 2.3 | 0.0 | 15.0 | 10.5 |  |
|  | Mixed | 110 | 2.1 | 0.0 | 25.9 | 7.9 |  |
|  | Woody wetland | 77 | 2.6 | 0.0 | 21.2 | 11.5 |  |

^a^Excluding aspen

^b^Weighted mean by proportion of each landcover within the study area

Table A3. Top N-mixture model for ruffed grouse drumming surveys each year as determined by AICc selection including estimates of detection and abundance with 95% confidence intervals (CI), Michigan’s Upper Peninsula, USA, 2013–2015.

| Year | Model^a^ | Detection estimate (%) | Abundance estimate^b^ | 95% CI |
| --- | --- | --- | --- | --- |
| 2013 | ~date ~asp | 24.5 | 178 | 93–346 |
| 2014 | ~date ~1 | 15.8 | 151 | 79–1246 |
| 2015 | ~date ~1 | 33.4 | 137 | 92–239 |

^a^N-mixture model includes covariates of detection on the left and abundance on the right. The “date” covariate was Julian date. The null model (intercept only) is indicated as “1”. Covariates for ruffed grouse include “asp” as the proportion of aspen (*Populus tremuloides* or *P. grandidentata*) as land cover within each survey site.

^b^Abundance estimates are for the audible area surveyed (550 m diameter with 65 sites for grouse) and only include estimates of abundance for drumming males in grouse surveys.

**APPENDIX B**

**Significant resource attributes from population-level resource utilization functions (RUF) for wolves and coyotes and k-fold cross-validation results for RUFs of wolves, coyotes, and white-tailed deer, Michigan’s Upper Peninsula, USA, 2013–2015.**

Table B1. Number of individuals that had significant (α < 0.05, confidence intervals do not include 0) positive (+) or negative (-) modeled relationship with each resource attribute from population-level resource utilization functions for wolves and coyotes (excluding land cover covariates). Resource utilization functions were estimated for active and inactive GPS locations during 3 time periods related to white-tailed deer: pre-parturition (PPP, 1–26 May), fawn limited mobility period (LMP, 27 May–30 June), and fawn social mobility period (SMP, 1 July–31 August), Michigan’s Upper Peninsula, USA, 2013–2015.

| Resource attribute | Coyote | | | | | | | | | | | | Wolf | | | | | | | | | | | |
| --- | --- | --- | --- | --- | --- | --- | --- | --- | --- | --- | --- | --- | --- | --- | --- | --- | --- | --- | --- | --- | --- | --- | --- | --- |
|  | Active | | | | | | Inactive | | | | | | Active | | | | | | Inactive | | | | | |
|  | PPP | | LMP | | SMP | | PPP | | LMP | | SMP | | PPP | | LMP | | SMP | | PPP | | LMP | | SMP | |
|  | + | - | + | - | + | - | + | - | + | - | + | - | + | - | + | - | + | - | + | - | + | - | + | - |
| Intercept | 1 | 4 | 4 | 6 | 3 | 8 | 0 | 3 | 1 | 4 | 0 | 6 | 1 | 3 | 3 | 4 | 7 | 4 | 0 | 0 | 2 | 2 | 2 | 3 |
| Distance to edge | 0 | 5 | 2 | 8 | 4 | 7 | 1 | 2 | 2 | 3 | 1 | 5 | 2 | 2 | 0 | 7 | 3 | 8 | 0 | 0 | 0 | 4 | 0 | 5 |
| Distance to road | NA | NA | NA | NA | 6 | 5 | 1 | 2 | 1 | 4 | 3 | 3 | 1 | 3 | 3 | 4 | 3 | 8 | 0 | 0 | 1 | 3 | 0 | 5 |
| Distance to water | 3 | 2 | 4 | 6 | 2 | 3 | NA | NA | 2 | 1 | NA | NA | 1 | 3 | 1 | 6 | 2 | 9 | 0 | 0 | 0 | 4 | 0 | 5 |
| Doe occurrence | 2 | 3 | 5 | 5 | 6 | 5 | NA | NA | NA | NA | 2 | 4 | 1 | 3 | 3 | 4 | 3 | 8 | 0 | 0 | 2 | 2 | 2 | 3 |
| Grouse density | 2 | 3 | 6 | 4 | 4 | 7 | 2 | 1 | 2 | 3 | 4 | 2 | 0 | 4 | 5 | 2 | 6 | 5 | 0 | 0 | 2 | 2 | 2 | 3 |
| Hare density | 2 | 3 | 5 | 5 | 6 | 5 | 0 | 3 | 2 | 3 | 4 | 2 | 4 | 0 | 5 | 2 | 6 | 5 | 0 | 0 | 4 | 0 | 2 | 3 |
| Patch size | NA | NA | NA | NA | 5 | 6 | NA | NA | 2 | 3 | 1 | 5 | NA | NA | 1 | 6 | NA | NA | NA | NA | NA | NA | NA | NA |
| Wolf occurrence | 3 | 2 | 6 | 4 | 6 | 5 | 1 | 2 | 2 | 3 | 2 | 4 | NA | NA | NA | NA | NA | NA | NA | NA | NA | NA | NA | NA |

Table B2. K-fold cross validation results for resource utilization functions for wolves, coyotes, adult female deer, and fawn deer during 3 time periods related to white-tailed deer: pre-parturition (PPP, 1–26 May), fawn limited mobility period (LMP, 27 May–30 June), and fawn social mobility period (SMP, 1 July–31 August), Michigan’s Upper Peninsula, USA, 2013–2015.

| Species | Activity | Period | Slope | R2 | Positive slope | Negative slope | Significant positive | Significant negative |
| --- | --- | --- | --- | --- | --- | --- | --- | --- |
| Wolves | Active | PPP | -2.02E-03 | 0.42 | 2 | 3 | 1 | 1 |
|  |  | LMP | 9.17E-03 | 0.62 | 10 | 1 | 8 | 0 |
|  |  | SMP | 1.16E-02 | 0.39 | 8 | 3 | 4 | 0 |
|  | Inactive | PPP | 4.93E-03 | 0.14 | 4 | 2 | 0 | 0 |
|  |  | LMP | 8.86E-03 | 0.53 | 11 | 0 | 7 | 0 |
|  |  | SMP | 7.30E-03 | 0.28 | 9 | 2 | 1 | 0 |
| Coyotes | Active | PPP | 5.15E-02 | 0.35 | 5 | 2 | 3 | 0 |
|  |  | LMP | 1.43E-03 | 0.54 | 7 | 6 | 5 | 2 |
|  |  | SMP | -7.41E-05 | 0.32 | 4 | 9 | 2 | 1 |
|  | Inactive | PPP | 6.20E-02 | 0.25 | 5 | 2 | 2 | 0 |
|  |  | LMP | 5.17E-04 | 0.38 | 8 | 5 | 4 | 1 |
|  |  | SMP | 6.84E-04 | 0.29 | 7 | 6 | 2 | 1 |
| Adult female deer | - | PPP | -3.14E-05 | 0.12 | 54 | 33 | 0 | 1 |
|  |  | LMP | 4.39E-03 | 0.49 | 72 | 17 | 41 | 6 |
|  |  | SMP | 1.54E-05 | 0.15 | 52 | 42 | 4 | 1 |
| Fawn deer | - | LMP | 4.95E-03 | 0.45 | 28 | 9 | 15 | 2 |
|  |  | SMP | 3.94E-03 | 0.54 | 34 | 3 | 20 | 0 |
